# A Digital Communication Intervention to Support Older Adults and Their Care Partners Transitioning Home After Major Surgery: Protocol for a Qualitative Research Study

**DOI:** 10.2196/59067

**Published:** 2024-08-28

**Authors:** Brian A Campos, Emily Cummins, Yves Sonnay, Mary E Brindle, Christy E Cauley

**Affiliations:** 1 Ariadne Labs Harvard TH Chan School of Public Health Boston, MA United States; 2 Department of Surgery Beth Israel Deaconess Medical Center Boston, MA United States; 3 Department of Pediatric Surgery University of Calgary Calgary, AB Canada; 4 Department of Surgery Massachusetts General Hospital Boston, MA United States

**Keywords:** mHealth, geriatric, surgery, care partner, qualitative study, digital intervention, intervention development

## Abstract

**Background:**

Older adults (aged ≥65 years) account for approximately 30% of inpatient procedures in the United States. After major surgery, they are at high risk of a slow return to their previous functional status, loss of independence, and complications like delirium. With the development and refinement of Enhanced Recovery After Surgery protocols, older patients often return home much earlier than historically anticipated. This put a larger burden on care partners, close family or friends who partner with the patient and guide them through recovery. Without adequate preparation, both patients and their care partners may experience poor long-term outcomes.

**Objective:**

This study aimed to improve and streamline recovery for patients aged ≥65 years by exploring the communication needs of patients and their care partners. Information from this study will be used to inform an intervention developed to address these needs and define processes for its implementation across surgical clinics.

**Methods:**

This qualitative research protocol has two aims. First, we will define patient and care partner needs and perspectives related to digital health innovation. To achieve this aim, we will recruit dyads of patients (aged ≥65 years) who underwent elective major surgery 30-90 days prior and their respective care partners (aged ≥18 years). Participants will complete individual interviews and surveys to obtain demographic data, characterize their perceptions of the surgical experience, identify intervention targets, and assess for the type of intervention modality that would be most useful. Next, we will explore clinician perspectives, tools, and strategies to develop a blueprint for a digital intervention. To achieve this aim, clinicians (eg, geriatricians, surgeons, and nurses) will be recruited for focus groups to identify current obstacles affecting surgical outcomes for older patients, and we will review current assessments and tools used in their clinical practice. A hybrid deductive-inductive approach will be undertaken to identify relevant themes. Insights from both clinicians and patient-care partners will guide the development of a digital intervention strategy to support older patients and their care partners after surgery.

**Results:**

This study has been approved by the Massachusetts General Hospital and Harvard Institutional Review Boards. Recruitment began in December 2023 for the patient and care partner interviews. As of August 2024, over half of the interviews have been performed, deidentified, and transcribed. Clinician recruitment is ongoing, with no focus groups conducted yet. The study is expected to be completed by fall 2024.

**Conclusions:**

This study will help create a scalable digital health option for older patients undergoing major surgery and their care partners. We aim to enhance our understanding of patient recovery needs; improve communication with surgical teams; and ultimately, reduce the overall burden on patients, their care partners, and health care providers through real-time assessment.

**International Registered Report Identifier (IRRID):**

DERR1-10.2196/59067

## Introduction

### Background

Older adults aged 65 years and older account for over 30% of the population of inpatients undergoing operations in the United States [1]. Older adults and their care partners typically begin their interaction with the surgical system as outpatients in an episodic fashion as they prepare for elective surgery. After surgery, the time spent in the hospital after many operations has shortened dramatically with the use of Enhanced Recovery After Surgery protocols [2]. Being prepared and informed prior to transitioning home is an important process that can improve patient and care partner’s confidence during their hospital stays and beyond. Unfortunately, communication during surgical discharge is often inadequate. Up to 80% of patients do not recall the information provided, 71% of nurses say they do not have enough time to meet patient engagement and education needs, and 50% of patients recall incorrect information [3,4]. Once home, older patients often depend on care partners, close family, or friends who partner with the patient and help guide them through recovery and who often take on this role with little or no training by the surgical care team.

This transition to home earlier in their recovery can be difficult for the older adult patient. Delirium [5,6], functional decline [7], and loss of independence [8,9] occur frequently in this patient population in the early postoperative period. These changes to a patient’s baseline function as well as sleep disturbance and medical burdens lead to anxiety and stress for patients and care partners, who are helping patients with activities of daily living during their recovery [10]. These compounded effects impact transitions to home and contribute to adverse outcomes, including mental health challenges, the need for hospital readmission, and loss of independence [11]. There is a critical need to address gaps in care quality to improve outcomes of older adults recovering from major surgery at home and support their care partners.

One possible and scalable way to address these gaps is through technology. It is a common misconception that most of the older population is resistant to technology; about 61% of adults aged ≥65 years owned a smartphone in 2021, and many more own a computer/laptop (90%) and use the internet (75%) [12,13]. Our current work strives to lay the groundwork for the development of a future digital health intervention to improve the outcomes of older adults and their care partners transitioning home by addressing unmet communication needs [14] and focusing on the perioperative period. We hypothesize that this qualitative study will yield critical information from all participants interviewed (patients, care partners, and clinicians) to create a useful digital health tool that addresses communication gaps and improves care coordination, thereby improving patient quality of life and reducing caregiver burden after surgery.

### Objectives

The overall goal of this study is to identify and understand the obstacles older adults and their care partners face while recovering at home after major surgery and to receive input from clinicians involved in their care regarding current geriatric conditions, assessments, and potential interventions. These findings will inform a future digital intervention that addresses shortcomings in current systems of care and is designed for older adults undergoing elective surgery along with their care partners.

## Methods

### Study Design

We will conduct an exploratory qualitative analysis to investigate the communication needs of patients, care partners, and clinicians and the potential role of a digital health tool to address these needs. We will conduct interviews with adults (aged ≥65 years) who have undergone major surgery as well as their identified care partners (through a separate 1:1 interview) to understand unmet needs and challenges they encountered during recovery. We will explore how they responded to these obstacles and the types of intervention they anticipate would be the most helpful. Subsequently, we will seek additional input from key clinical stakeholders, specifically geriatricians, surgeons, and nurses, to explore the assessment tools and approaches that they feel would support the needs of older patients and their care partners during postoperative recovery and specifically how a digital tool could facilitate that support. A hybrid deductive-inductive approach will be undertaken to identify relevant themes [15]. Results will be used to inform the future development of a digital solution to support these patients after surgery and their care partners.

### Inclusion and Exclusion Criteria

#### Patients and Care Partners’ Semistructured Interviews

Surgical clinic patients from an urban academic medical center and community surgical center who underwent colorectal, orthopedic, and thoracic surgery will be considered for inclusion. To be included, patients must also be 65 years or older, have undergone recent (within 90 days) elective major surgery, and speak English. As for their care partners, they will be 18 years or older and be English speakers. Both patients and their respective care partners will need to be recruited (a dyad) for inclusion in the study. Exclusion criteria include cognitive impairment causing an inability to perform teach-back for consent to interview, as outlined in prior National Institutes of Health–funded studies [16]. If one-half of the dyad does not complete the requirements of the study, the other half’s information and data will not be included in the study analysis.

#### Clinician Focus Groups

We will include English-speaking health care workers within the clinician focus groups. These participants will be derived from the following populations nationwide: geriatricians, surgeons, anesthesiologists, advanced practice providers, physical therapists or occupational therapists, social workers or case managers, and nurses with experience with older adults recovering from surgery. Exclusion criteria include having less than 1 year of clinical experience, having no or limited experience with the older adult surgical patient population, and practicing outside of the United States.

### Recruitment and Sampling

#### Patients and Care Partners’ Semistructured Interviews

Older adults presenting to the Massachusetts General Hospital or North Shore Medical Center Surgical clinics (both part of the Mass General Brigham integrated health system) and their care partners meeting eligibility criteria will be recruited. The research team will query the operative list from the colorectal, thoracic, and orthopedic surgery patient population.

Eligible patients will be approached by the research fellow, who will review the study purpose, eligibility criteria, and informed consent documents using standard processes. Purposive sampling will ensure a diverse group of older surgical patients, with the inclusion of patients aged 80 years or older, to capture patients across an age spectrum [17]. In addition, the team will partner with the Massachusetts General Hospital Community Access, Recruitment, and Engagement Center to recruit patients from minority communities and those with low socioeconomic status (ie, education less than a high school diploma) to ensure the evaluation of recovery needs among a diverse patient population.

We expect to recruit 5 patients and their care partners from each of the 3 specialties, with a total of 30 interviews to reach saturation [18].

#### Clinician Focus Group Recruitment

Among clinicians currently practicing in the United States, we will recruit a nationally representative sample of clinician participants by email in which we outline the study purpose and attach the consent form for participation in focus groups. We will leverage personal contacts in nursing, surgery, and geriatrics as well as national professional society meetings (such as the American Society of Colon and Rectal Surgeons, American Geriatrics Society, and American College of Surgeons) to obtain email addresses and optimize recruitment. Once participants are identified, we will schedule focus groups aligned by specialty to optimize group dynamics and encourage psychological safety as described in similar studies. We will plan to recruit 8 clinicians per group to account for nonattendance. We will conduct interviews and focus groups until thematic saturation is reached, with an initial estimate of 24 participants across 4 focus groups needed based on past studies and practical limitations of available clinicians [19,20]. Focus groups of around six members have been described as optimal for meaningful discussion [21].

### Ethical Considerations

Ethical approval was obtained from the Harvard TH Chan School of Public Health Institutional Review Board (IRB23-0790). Survey information is collected and saved securely on REDCap (Research Electronic Data Capture). Informed consent is obtained prior to starting the interviews. Participants are informed of any intent to record prior to the start of the recording. Recordings are then transcribed, deidentified, and uploaded to a secure server. For each patient and care partner who completes both the survey and interview, they will receive US $20 for their efforts. Concerted efforts are made to purposefully recruit patients from varying sociodemographic, cultural, and ethnic backgrounds to obtain diverse perspectives from patients across surgical subspecialties.

### Interviews

#### Patients and Care Partners’ Semistructured Interviews

Prior to the qualitative interview, we will send out an electronic survey via email through REDCap to both the patient and designated care partner; each participant will answer demographic and quality of life questions (the latter is based on the World Health Organization Quality of Life questionnaire [WHOQOL-BREF]) [22,23]. The care partner will answer additional questions based on the Zarit Burden Interview [24,25]. These survey emails take 15 minutes or less to complete. The WHOQOL-BREF questionnaire is scored based on a predefined scoring system that is then calculated into domain scores, which range from 0 to 100, with a higher score representing a better health state. Each question on the Zarit Burden Interview is scored based on the Likert scale, with a higher composite number representing a greater burden to the care partner. Both questionnaires are highly validated tools that have been used for decades [23,25].

During the virtual or phone qualitative interview, we will (1) characterize patients’ and care partners’ perceptions of the surgical experience and how they cope with its challenges; (2) identify appropriate intervention targets, such as the 5 Ms of geriatrics (ie, mind, mobility, medications, what matters most, and multicomplexity); (3) assess patients’ and care partners’ preferences for the structure or mode of delivery (including video, phone, in-person, or via an application or desktop platform), the timing of the intervention, and the number of sessions; (4) review potential module content and the resources used; and (5) assess patients’ and care partners’ perception about their usefulness (Multimedia Appendices 2 and 3).

#### Clinician Focus Groups

Prior to the qualitative interview, clinicians will fill out an electronic survey via REDCap that will capture demographic data. During the virtual focus groups, we will (1) evaluate geriatric conditions affecting outcomes from major surgery among older adults, (2) discuss geriatric assessments performed to evaluate these needs, (3) rate assessments and tools, and (4) discuss procedures for intervention delivery (Multimedia Appendix 4).

Remote interviews will be conducted using Health Insurance Portability and Accountability Act–compliant video conference software (eg, Microsoft Teams; version 24004.1304.2655.7488). We will use audio and video to record the interviews and transcribe them.

### Analysis

Interviews will be coded using a multistep approach [26,27]. First, 2 members of the research team will create an a priori codebook, drawing codes directly from the interview guide. A priori themes will include (1) presurgery experiences, (2) clinical care team communication, (3) postsurgical challenges, and (4) description and use of a digital intervention. A set of 2 transcripts will be coded using this codebook. At least 2 members of the research team will then meet to discuss and resolve discrepancies and update the codebook accordingly. Next, at least 2 members of the research team will inductively code 2 additional transcripts to identify additional themes not included in the a priori codebook. Based on this open coding, inductive codes will be added to the codebook. All transcripts will be coded against this codebook. All coding will be conducted in the NVivo 20 software. At least 2 members of the research team will code all transcripts, meeting periodically to discuss discrepancies and ensure a consensus is reached. NVivo software’s coding comparison feature will be used to compare coding and identify any major discrepancies that emerge in the coding process. We will compare each researcher’s coding across all transcripts and resolve discrepancies on a regular basis until all coding is complete [28].

Clinician, patient, and care partner interviews and focus groups will guide intervention development. Our research team will meet weekly to discuss the analytic plan and intervention development.

## Results

This study is funded by the Agency for Healthcare Research and Quality grant (R01HS029454). The study was approved by the institutional review board of the Massachusetts General Hospital and Harvard. Recruitment began in December 2023 for the patient and care partner interviews. As of August 2024, over half of the proposed patient-care partner interviews have been performed, deidentified, and transcribed. A proposed codebook has been developed and the coding process is underway but not yet completed. Clinicians are currently being recruited and no focus groups have been performed yet.

## Discussion

### Expected Outcomes

The overall goals of this qualitative study are to identify and understand the obstacles older adults, care partners, and clinicians face when transitioning home after major surgery and to use this understanding to inform a potential intervention that addresses the needs of these groups during this critical time. This protocol will outline stakeholder perspectives on clinically important geriatric conditions, assessments, and interventions to meet the currently unmet needs of this growing patient population. The results of these semistructured interviews and focus groups will inform the development of a digital health intervention aimed at improving the quality of life for patients and reducing care giver burden during surgical recovery by addressing communication gaps and current shortcomings in care coordination.

Given the increasing use of the internet and mobile-based technologies for delivering health care during and following the COVID-19 pandemic [12], it is imperative that clinicians and surgical systems leverage digital health applications to improve transitions of care, particularly for vulnerable populations, including older adults [29]. For example, if participants identify physical and neurocognitive decline after major surgery as a challenge for patients, the digital intervention we develop could use personalized, timely assessments to identify and address these findings to optimize care for older adults transitioning home after surgery [30]. If participants note challenges with coordinating access to care after surgery (eg, presenting to the emergency room versus the surgical clinic for wound concerns), we can develop tailored communication to improve care coordination through the intervention. In addition, timely anticipatory guidance based on participant feedback can ensure patients and care partners prepare their homes and obtain supplies helpful during their recovery. By designing the intervention with input from both academic and community surgical practices, we propose to develop an intervention that is acceptable, usable, and based on the best evidence. This design process should ensure that the resulting tool will be readily adopted by a wider audience.

Despite the burgeoning use of technology to facilitate remote health monitoring, older adults are sometimes excluded from the benefits that these technologies provide [31]. Lack of access to health care–related technology is a barrier to adoption and is influenced greatly by both exposure and cost [32]. The social and professional environment and personal preferences of older adults may not inspire or influence them to follow and adopt this technology as readily as their younger counterparts. It is often the role of the younger care partner or family member to learn and then teach older adults how to use newer technologies. If the older adult does not have access to this information (by owning the necessary technology or having the baseline skills required of digital literacy, such as using a computer or smartphone) or someone to teach them (a care partner who has this knowledge or access to newer technologies), then the patient will often resort to a more traditional or antiquated alternative. These new technologies can also be cost prohibitive, especially for older adults who have limited resources. Buying the latest gadget or health monitor may not be in their budget [33].

Another obstacle is that digital health options are often designed for young populations with excellent vision and manual dexterity in addition to advanced digital literacy [34]. It is not uncommon for older adults to require large and bolded text on their phones because of the loss of visual acuity. In addition to small text, many electronic applications have inappropriately colored or contrasted text that older adults have trouble reading [35]. In addition, these devices and applications may have functions that may rely on a preexisting familiarity with the technology’s functionality unfamiliar to older adults not fully immersed in the digital ecosystem.

This qualitative study will strive to consider all these diverse factors. If an interview participant does not have internet access or knowledge of videoconferencing, then a phone interview will be conducted. It will gauge patient and care partner challenges and their current solutions. It will identify the resources they had (before and after surgery) and those they wished they had. It will assess current effective and unproductive modes of communication. Most importantly, it will gauge their direct sentiments about a digital health option that could potentially address the shortcomings identified in their interview answers. This protocol will ensure that any digital health option generated in the future will take into account older adults’ perspectives as well as those of their care partners and clinicians. This will minimize waste, ensure the tools developed are designed within the targeted audience’s physiological and biopsychosocial restrictions, and increase the probability of adoption and utility. Importantly, this study will also explore how digital technologies can be used by the adjacent care partner to support an older adult during recovery. Care partners may have differing needs from the patients and different degrees of engagement with digital technology. This study may ultimately identify opportunities for novel digital health capabilities through technology that links the patient-care partner dyad with the health care team.

### Strengths and Limitations

There are several strengths to this study. This is one of the first studies to evaluate communication barriers of the older adult population undergoing major surgery and explore how a digital intervention might improve transitions home after surgery. It will also consider the care partner perspective, who may be of the same generation (oftentimes spouses or close relatives). The study will include diverse clinician perspectives, particularly those professionals who interact with older patients frequently. This information can then be leveraged to develop a prototype digital health intervention that is useful and acceptable using a human-centered design approach. The application will then undergo refinement to further improve acceptability. Smartphone-based education and remote self-monitoring are scalable solutions that can improve outcomes in this growing high-risk patient population. This protocol outlines the foundational development of a future digital health solution to support the needs of older adults undergoing major surgery as well as the needs of their care partners. The findings from this work can be scaled and spread to increase the value of care provided for a variety of health conditions and will drive future efforts to improve transitions home after major surgery, particularly for current and future generations of older adults—by the end of this decade a fifth of Americans will be aged older than the 65 years [36].

Limitations of this study include potential limits in generalizability based on our recruitment of patients from the geographic northeast of the United States. To overcome this limitation, we will intentionally recruit a diverse patient population. Another limitation of this study is that all patients and care partners will be English speakers. Future work will need to address this shortcoming with multilingual qualitative interviewers to culturally adapt content of the planned intervention. We will include patients from 3 major surgical specialties that service a large older adult patient population. Although this study is based in the United States, it reflects the same concerns addressed in countries globally that are looking to adopt digital health technology. Our findings may have generalizability to other countries that are comparable in terms of economic development and digital technology adoption. We will also strive to include the perspective of patients and care partners with low income and those below the poverty threshold and reflect on the particular limitations encountered by these groups. It will be important to note the possibility that benefits derived from future interventions based on this work may deepen the digital divide that already exists, as it can leave behind those who, for financial, health, or geographic reasons, cannot benefit from more real-time assessment [37,38]. Possible solutions proposed have been for governments to subsidize basic or newer technologies [39] to facilitate adoption and to be purposeful when building new or reinforcing internet infrastructures with a focus on marginalized populations [40].

### Conclusions

This qualitative research protocol will improve our understanding of the obstacles older adults and their care partners face while recovering at home after major surgery. The described approach will yield critical data to inform the development of a digital health intervention that is tailored to all those involved in the care of the older adult surgical population with a particular focus on care partners that are often provided limited support from the surgical care team. These findings will inform a future digital intervention that addresses shortcomings in current systems of care and will be designed to support older adults undergoing major surgery, their care partners, and clinician team members.

## Appendix

Multimedia Appendix 1 Peer-review reports from the Healthcare Information Technology Research (HITR) - Agency for Healthcare Research and Quality (USA).

Multimedia Appendix 2 Questions to be asked during the patient qualitative interview.

Multimedia Appendix 3 Questions to be asked during the care partner qualitative interview.

Multimedia Appendix 4 Questions to be asked during the clinician focus group interview sessions.

## Abbreviations

REDCap: Research Electronic Data Capture
WHOQOL-BREF: World Health Organization Quality of Life questionnaire

## Data Availability

The data sets generated and analyzed during this study are available from the corresponding author on reasonable request.
