# Supplementary material for: A Digital Communication Intervention to Support Older Adults and Their Care Partners Transitioning Home After Major Surgery: Protocol for a Qualitative Research Study
Source: JMIR Res Protoc. 2024 Aug 28;13:e59067. doi: 10.2196/59067 (PMC11391150; doi:10.2196/59067)
Supplement: Multimedia Appendix 1 [file resprot_v13i1e59067_app1.pdf]

SUMMARY STATEMENT
(Privileged Communication)

PROGRAM CONTACT:
Amy Lindinha
(301) 427-1614
amy.lindinha@ahrq.hhs.gov

Release Date: 03/06/2023
Revised Date:

Application Number: 1 R01 HS029454-01

Principal Investigator

BRINDLE, MARY ELIZABETH

Applicant Organization: HARVARD SCHOOL OF PUBLIC HEALTH

Review Group: HITR
Healthcare Information Technology Research

Meeting Date: 02/23/2023
Council: MAY 2023
Requested Start: 07/01/2023
RFA/PA: PA20-068
PCC: CEPI

Project Title: Scalable Digital Communication Intervention to Support Older Adults and Care-partners Transitioning Home After Major Surgery
SRG Action: Impact Score:36 Percentile:15 +

Human Subjects: 30-Human subjects involved - Certified, no SRG concerns
Animal Subjects: 10-No live vertebrate animals involved for competing appl.
Gender: 1A-Both genders, scientifically acceptable
Minority: 1A-Minorities and non-minorities, scientifically acceptable
Clinical Research - not NIH-defined Phase III Trial

Project Year	Direct Costs Requested	Estimated Total Cost
1	212,170	401,660
2	211,787	400,935
3	207,946	393,663
4	213,363	403,918
5	210,802	399,070
TOTAL	1,056,068	1,999,245

ADMINISTRATIVE BUDGET NOTE: The budget shown is the requested budget and has not been adjusted to reflect any recommendations made by reviewers. If an award is planned, the costs will be calculated by Institute grants management staff based on the recommendations outlined below in the COMMITTEE BUDGET RECOMMENDATIONS section.

RESUME AND SUMMARY OF DISCUSSION: This R01 health services research grant application from Dr. Mary Brindle, from Harvard School of Public Health, in Boston, MA, proposes a project to develop and refine of the myPOSH intervention, a digital health solution to support the needs of older adults undergoing major surgery and their care-partners. The reviewers agreed on the significance of this project noting that older adults account for over forty percent of inpatient operations and over one third of outpatient procedures performed annually across the United States. Being informed prior to transitioning home is an important process that can improve patient and care-partner's confidence during their hospital stay and as they transition home. The reviewers added that there is a critical need to address point of care in transition to home in elderly population and to support their care partners. Findings from this work can be scaled and spread to increase the value of care provided for various health conditions and will drive future efforts to improve transitions home after major surgery. The overall strategy, methodology, and analyses are well-reasoned and appropriate to accomplish the specific aims of the project. The application includes a strong preliminary studies section demonstrating the case for the use of digital tools to improve care for older adults during transitions from surgery to home in for patients undergoing major surgeries for multiple conditions. The application also includes relevant use of theory to frame the development and assessment of MyPOSH. The assessment domains are informed by the Geriatric 5 Ms: Mind, Mobility, Medications, What Matters Most, and Multicomplexity. However, the reviewers had some concerns. The application does not show high innovation in clinical practice or approaches to care. The innovation might be considered in the patient population being served by the myPOSH tool. The Principal Investigator (PI) is listed at only 10% effort. Subaward PI listed with an average of just above 10% per year, but in year 1 only listed at 0.89% which falls below a combined 20% for both PIs in year 1. In addition, there is no evidence of prelim/pilot data of the app. Based on the Scientific and Technical Merit of the application, this application received a score of 36.

DESCRIPTION (provided by applicant): In this R01 proposal, we outline a comprehensive 5-year research proposal that will support development and rigorous testing of a scalable intervention to improve integration of geriatric principles into surgical care of older adults with the goal of improving patient and care-partner quality of life from transitions into and out of the hospital around the time of surgery. This significant and innovative plan aims to improve care transitions for this high risk patient population as they are discharged home after major surgery. Background: Surgeons try to prepare and support older adults through the perioperative transitions to the hospital and back home, with variable success. Most surgeons are not fully equipped to address geriatric needs that exist among the rising number of older adults presenting to surgical attention. Addressing health outcomes that older adults prioritize when considering surgery is an important opportunity to improve surgical care quality in this growing patient population through development of novel interventions. Specific aims and research design: We aim to develop the first version of myPOSH, a digital intervention to improve the care of older patients undergoing surgery including development of content and application with methodology for implementation through 1) interviews with older adults undergoing major surgery and their care-partners when available (up to n=30) (Aim 1.1), with additional input from 2) key clinical stakeholder focus groups (i.e., surgeons, geriatricians, and nurses) (n=4; at least 24 clinicians) (Aim 1.2). Next, we will examine the preliminary feasibility and acceptability of myPOSH via an open pilot (n=20 patients, n=2 surgical teams across 2 sites) with exit interviews and pre-post assessments. myPOSH will be refined (Aim 2). Finally, we will examine the feasibility and acceptability of myPOSH versus usual surgical care (n=84 patients; 42 patients per arm) in a pilot RCT following predetermined benchmarks (Aim 3). These findings will inform a hybrid efficacy trial through future funding with plans to extend this work to other surgical clinic sites. Relevance: This R01 is in line with the goal of this funding announcement to develop scalable interventions to improve care transitions among high risk patient populations. Impact: As surgeons with expertise in surgical systems change, we recognize the critical need to improve surgical care quality among older adults transitioning home after surgery. Experiences

gained through this proposal are foundational to future research to improve caregiver preparedness and reduce patient anxiety in the perioperative setting.

PUBLIC HEALTH RELEVANCE: Smartphone-based education and remote self-monitoring are scalable solutions that can address unmet information needs and address poor care coordination, anxiety, and caregiver burden among older adults transitioning home after major surgery. This proposal outlines the development and refinement of the myPOSH intervention, a digital health solution to support the needs of older adults undergoing major surgery and their care-partners. The findings from this work can be scaled and spread to increase the value of care provided for a variety of health conditions and will drive future efforts to improve transitions home after major surgery.

CRITIQUE 1

Significance:	2
Investigator(s):	2
Innovation:	5
Approach:	3
Environment:	1

Overall Impact: The goal of this study is to improve care outcomes of older adults and their care-partners (family/loved one who partners in care) transitioning home after major surgery by addressing unmet communication needs. Per the application, people over the age of 65 account for over 40% of inpatient operations performed in the United States. These patients increasingly transition home from hospital earlier in their recovery and can experience negative outcomes such as delirium, functional decline, and loss of independence. When combined with sleep disturbance and medical burdens, these issues cause anxiety -- compounding the strain on family and care-partners. These compounded effects impact transitions home and contribute to adverse outcomes including poor long-term survival. The application seeks to develop a tool to act as a virtual coach to prepare and support older patients and care-partners transitioning home after major surgery. The investigator team and study environment are strong. The application presents a study aimed at addressing a significant issue, however the innovation of the project is not particularly high. There are some minor issues in the approach section.

1. Significance:
Strengths
- Older adults account for over 40 percent of inpatient operations and over one third of outpatient procedures performed annually across the United States. This number is expected to rise as the population age. Recovering from major surgery can be stressful for older adults and their care-partners due to negative outcomes that they may experience including delirium, functional decline and loss of independence.
- Being informed prior to transitioning home is an important process that can improve patient and care-partner's confidence during their hospital stay and as they transition home.
- From the application, communication during surgical discharge is often inadequate. Up to 80% of patients do not recall information provided, 71% of nurses say they do not have enough time to meet patient engagement and education needs, and 50% of patients recall information that is incorrect.

Weaknesses
- None noted.

2. Investigator(s):
Strengths
- The PI, collaborators, and other researchers well suited to the project.
- The investigators have complementary and integrated expertise, including experience relevant to implementation in clinical settings.
- are their leadership approach, governance and organizational structure appropriate for the project?
- The investigators have demonstrated ongoing records of accomplishments that have advanced their field(s).

Weaknesses
- PI listed at only 10% effort. Subaward PI listed with an average of just above 10% per year, but in year 1 only listed at 0.89% which falls below a combined 20% for both PIs in year 1.

3. Innovation:
Strengths

- The application seeks to address a gap in communication during care transitions for older adults, easing care-person burden and improving patient outcomes post-surgery.

Weaknesses
- The application does not show high innovation in clinical practice or approaches to care. The innovation might be considered in the patient population being served by the myPOSH tool.

4. Approach:
Strengths
- The overall strategy, methodology, and analyses are well-reasoned and appropriate to accomplish the specific aims of the project.
- The application includes a strong preliminary studies section demonstrating the case for the use of digital tools to improve care for older adults during transitions from surgery to home in for patients undergoing major surgeries for multiple conditions.
- Relevant use of theory to frame the development and assessment of MyPOSH. The assessment domains are informed by the Geriatric 5 Ms: Mind, Mobility, Medications, What Matters Most, and Multicomplexity.
- Strong qualitative methods section in Aim 1, including the use of theory in data collection and analysis and a description of a hybrid deductive – inductive approach to analysis.
- Aims 2 and 3 are well described.
- The project includes a detailed project timeline.
- The application includes a strong dissemination plan.
- Inclusion/exclusion criteria are justified in the research plan.
- Overall, the investigators present strategies to ensure a robust and unbiased approach.

Weaknesses
- Occasionally interviews are used interchangeably with focus groups in the methods section and other places of the application; however, this is only a minor weakness.
- More detail is needed on how the study will assess for differences in usual care (UC), and how those differences will be treated during analysis.
- For aim 3, it is not clear in the application who will provide the training to surgeons and clinical staff.

- No plans to make sure surgeons and clinical staff who participate in Aim 2 acceptability study are not also used in Aim 3 for the feasibility study.

5. Environment:
Strengths
- The scientific environment in which the work will be done is strong and will contribute to the probability of success
- Letters of support are provided and show institutional support for the project.

Weaknesses
- None noted.

Additional Review Criteria:

How well does the proposed research plan align with AHRQ's mission and research priorities?
Acceptable

Does the approach focus on rigorously testing promising interoperable interventions that improve care transitions?
Acceptable/Yes

Is the usability of the intervention evaluated?
Acceptable/Yes

Does the approach measure interoperability, relevant patient outcomes and reductions in adverse events?
Acceptable

Is the intervention designed with sustainability and scalability in mind?
Acceptable/Yes

Is the dissemination plan well-described?
Acceptable/Yes

Protections for Human Subjects: Acceptable.

Data and Safety Monitoring Plan: Acceptable.

Single IRB for Cooperative Research: Acceptable.

Inclusion of Women: Acceptable.

Inclusion of Minorities: Acceptable.
- English speaking is an inclusion requirement.

Inclusion of Priority Populations: Acceptable.

Degree of Responsiveness: Responsive.

Additional Review Considerations:

Data Management Plan: Adequate.

Budget and Period of Support: Acceptable.

CRITIQUE 2

Significance:	4
Investigator(s):	1
Innovation:	4
Approach:	4
Environment:	1

Overall Impact: Overall, they intend to improve older adult discharge planning to home following major surgery. he intended digital intervention ("myPOSH") will be investigated through a usability and feasibility study that progresses to a pilot RCT. There is a critical need to address point of care in transition to home in 65+ population and to support their care partners. Essentially, it is smartphone-based education and remote self-monitoring, which is not necessarily novel, but could meaningfully contribute to the science because of the attention to the patient-caregiver dyad.

1. Significance:
Strengths
- The PI has experience with smartphone interventions.
- Theoretical framework is Geriatric 5 MS: Mind, Mobility, What Matters Most, and Multicomplexity (Nagy, et al. 2021)
- Reasonable intent to use this R01 Mechanism to evaluate feasibility and usability before launching a fully powered, multisite efficacy trial of the app versus usual surgical care.

Weaknesses
- No evidence of prelim/pilot data of the app.

2. Investigator(s):
Strengths
- Collaborators are well-prepared and suited for this work (Harvard School of Public Health; Mass General; and a sub-award to Aptima (needed for software).
- The Principal Investigator is the Director of the Safe Surgery Program at Ariadne Labs

Weaknesses
- PI offers only 10% (overall coordination) while Co-I is at 15% (coordination, recruitment of surgical patients, study procedures).

3. Innovation:
Strengths
- Could advance our understanding of point of care post-op discharge on the dyad (patient-caregiver).

Weaknesses
- Quality of life is very difficult to measure because of context and meaning of "quality" for each person. The concept of QOL is represented in the biosketches and references, but there is no

conceptual model or theoretical framework. Really, just a definition of QOL for this specific problem would be helpful.

4. Approach:
Strengths
- Acceptable and typical usability & feasibility study that progresses to a small RCT.
- Rigor is addressed through the NIH stage model to guide development and refinement
- Timeline is acceptable.

Weaknesses
- Would like prelim/pilot data.

5. Environment:
Strengths
- Aptiva for software.
- MGH for clinical trial.

Weaknesses
- None noted.

Additional Review Criteria: All Acceptable.

Protections for Human Subjects: Acceptable.

Data and Safety Monitoring Plan: Acceptable.

Single IRB for Cooperative Research: Acceptable.

Inclusion of Women: Acceptable.

Inclusion of Minorities: Acceptable.

Inclusion of Priority Populations: Acceptable.

Degree of Responsiveness: Responsive.

Data Management Plan: Adequate.

Budget and Period of Support: Seems appropriate, includes post doc. Appreciated the travel information so we could see per diem rates for Mass.

CRITIQUE 3

Significance:	3
Investigator(s):	2
Innovation:	3
Approach:	4
Environment:	1

Overall Impact: This R01 proposal, led by Dr. Brindle of Harvard School of Public Health, to respond to the PA's call to develop scalable interventions to improve care transitions among high-risk patient populations. The investigators want to develop and test a scalable intervention to improve integrating geriatric principles into surgical care of older adults to improve patient and care-partner quality of life from transitions into and out of the hospital around the time of surgery. Aim 1: Define the unmet education and communication needs of older adults and their care-partners when preparing for and recovering from transitions between the hospital and home to create a scalable solution. Aim 2: Evaluate usability and acceptability of the myPOSH intervention in two surgical clinics to refine the intervention while considering scalability across surgical settings. Aim 3: Determine feasibility of a multi-site RCT of the myPOSH intervention relative to usual surgical care (UC). Overall, the application is well-developed and well-written, with minor issues.

1. Significance:
Strengths
- Development and refinement of the myPOSH intervention, a digital health solution to support the needs of older adults undergoing major surgery and their care partners.
- Findings from this work can be scaled and spread to increase the value of care provided for various health conditions and will drive future efforts to improve transitions home after major surgery.

Weaknesses
- Mobile apps aren’t innovative anymore other than the patient population what innovation is there?

2. Investigator(s):
Strengths
- The investigators are well-suited to execute this project.
- The PIs have previously collaborated. They have complementary expertise and shared publications.

Weaknesses
- No major weaknesses

3. Innovation:
Strengths
- Multidisciplinary input for app development.
- The app being scalable.

Weaknesses
- Mobile apps aren’t innovative anymore other than the patient population what innovation is there?

4. Approach:
Strengths
- Focus groups to gain information is a good practice.
- Multidisciplinary input.

Weaknesses
- Aim 1: will the patients and care partners be matched? Are you recruiting 5 colectomy, 5 hip replacement, and 5 lung resections?
- Geriatricians, surgeons, anesthesiologists, APP, PT/OT, SW/CM, and nurses with experience with older adults recovering from surgery: It may not be important how many from each

discipline. The target focus group is 6 members. How are you going to ensure a balanced response to your recruitment?
- 60 minutes can be a long time will there be incentives?

5. Environment:
Strengths
- Environments are well-resourced and have significant research endeavors.
- Letters of support are provided and show support of the project.

Weaknesses
- No major weaknesses.

Protections for Human Subjects: Acceptable.

Data and Safety Monitoring Plan: Acceptable.

Single IRB for Cooperative Research: Acceptable.

Inclusion of Women: Acceptable.

Inclusion of Minorities: Acceptable.

Inclusion of Priority Populations: Acceptable.

Degree of Responsiveness: Acceptable.

Data Management Plan: Adequate.

Budget and Period of Support: Acceptable.

THE FOLLOWING SECTIONS WERE PREPARED BY THE SCIENTIFIC REVIEW OFFICER TO SUMMARIZE THE OUTCOME OF DISCUSSIONS OF THE REVIEW COMMITTEE, OR REVIEWERS' WRITTEN CRITIQUES, ON THE FOLLOWING ISSUES:

PROTECTION OF HUMAN SUBJECTS: ACCEPTABLE

INCLUSION OF WOMEN PLAN: ACCEPTABLE

INCLUSION OF MINORITIES PLAN: ACCEPTABLE

INCLUSION ACROSS THE LIFESPAN: ACCEPTABLE

COMMITTEE BUDGET RECOMMENDATIONS: The budget was recommended as requested.

Footnotes for 1 R01 HS029454-01; PI Name: Brindle, Mary Elizabeth

+ Derived from the range of percentile values calculated for the study section that reviewed this application.

MEETING ROSTER

Healthcare Information Technology Research
AGENCY FOR HEALTHCARE RESEARCH AND QUALITY
HITR
02/23/2023 - 02/24/2023

CHAIRPERSON(S)

JENDERS, ROBERT ALLEN, MD
PROFESSOR OF MEDICINE AND SENIOR ASSOCIATE DIRECTOR
CLINICAL AND TRANSLATIONAL SCIENCE INSTITUTE
UNIVERSITY OF CALIFORNIA, LOS ANGELES
LOS ANGELES, CA 90059

MEMBERS

ASAN, ONUR, BS, MS, PHD
ASSOCIATE PROFESSOR
SCHOOL OF SYSTEMS AND ENTERPRISES
STEVENS INSTITUTE OF TECHNOLOGY, NEW JERSEY
1 CASTLE TERRACE POINT
HOBOKEN, NJ 07030

BUCHER, BRIAN T, MD
DEPARTMENT OF SURGERY
UNIVERSITY OF UTAH SCHOOL OF MEDICINE
SALT LAKE CITY, UT 84113

CANTOR, MICHAEL, MA, MD
VICE PRESIDENT
CLINICAL INFORMATICS
REGENERON GENETICS CENTER
TARRYTOWN, NY 10591

CARON, ALEECE, MA, PHD *
CO-DIRECTOR OF EDUCATION & ASSOCIATE PROFESSOR OF MEDICINE
THE POPULATION HEALTH RESEARCH INSTITUTE
THE METROHEALTH MEDICAL CENTER
CASE WESTERN RESERVE UNIVERSITY
CLEVELAND, OH 44109

CHUI, MICHELLE ANNE, PHD, PHMD
HAMMEL SANDERS PROFESSOR
SOCIAL & ADMINISTRATIVE SCIENCES
SCHOOL OF PHARMACY
UNIVERSITY OF WISCONSIN
MADISON, WI 53705

EMBI, PETER J., MD, MS, BS *
PRESIDENT/CEO, REGENSTRIEF, INC.
SAM REGENSTRIEF PROFESSOR OF MEDICINE
ASSOCIATE DEAN FOR INFORMATICS & HEALTH SERVICES RESEARCH
IU SCHOOL OF MEDICINE
INDIANAPOLIS, IN 46202

KOSZALINSKI, REBECCA SUSAN, PHD, MS, BSN
AFFILIATE RESEARCH ASSOCIATE PROFESSOR
CHRISTINE E. LYNN COLLEGE OF NURSING
FLORIDA ATLANTIC UNIVERSITY
BOCA RATON, FL 33431

LANHAM, HOLLY J., PHD, MBA *
ASSOCIATE PROFESSOR & SCIENTIFIC DIRECTOR
DEPT. OF PSYCHIATRY & BEHAVIORAL SCIENCES
TEXAS MEDICATION FOR OPIOID USE DISORDER
UNIVERSITY OF TEXAS HEALTH SAN ANTONIO
SAN ANTONIO, TX 78229

MELTON, BRITTANY LEE, PHD, PHMD, BS *
ASSOCIATE PROFESSOR
PHARMACY PRACTICE
UNIVERSITY OF KANSAS
KANSAS CITY, KS 66160

MOONEY, SEAN DAVID, PHD
PROFESSOR
BIOMEDICAL INFORMATICS AND MEDICAL EDUCATION
UNIVERSITY OF WASHINGTON
SEATTLE, WA 98109

MOYE-DICKERSON, PAMELA, PHMD
CLINICAL ASSOCIATE PROFESSOR
PHARMACY PRACTICE/COLLEGE OF PHARMACY
MERCER UNIVERSITY
ATLANTA, GA 30341

NGUYEN, HIEN VAN, PHD
ASSISTANT PROFESSOR
DEPARTMENT OF ELECTRICAL AND COMPUTER ENGINEERING
UNIVERSITY OF HOUSTON
HOUSTON, TX 77204

NING, XIA, PHD
ASSOCIATE PROFESSOR
BIOMEDICAL INFORMATICS
COMPUTER SCIENCE AND ENGINEERING,
OHIO STATE UNIVERSITY
COLUMBUS, OH 43210

NOVAK, LAURIE L, PHD, MHSA, BA

RAO, GOUTHAM, MD
PROFESSOR AND CHAIRMAN
FAMILY MEDICINE & COMMUNITY HEALTH
CASE WESTERN RESERVE UNIVERSITY &
UNIVERSITY HOSPITALS OF CLEVELAND
CLEVELAND, OH 44106

ROSSETTI, SARAH COLLINS, PHD, RN
ASSISTANT PROFESSOR OF BIOMEDICAL INFORMATICS
AND NURSING
DEPARTMENT OF BIOMEDICAL INFORMATICS
SCHOOL OF NURSING
COLUMBIA UNIVERSITY MEDICAL CENTER
NEW YORK, NY 10032

SORONDO, BARBARA MIRNA, MBA, MD
DIRECTOR
CLINICAL DEVELOPMENT
LG CHEM LIFE SCIENCES INNOVATION CENTER, INC.
CAMBRIDGE, MA 02142

TABER, DAVID J., PHMD, MS *
PROFESSOR
DEPARTMENT OF SURGERY
DIVISION OF TRANSPLANT SURGERY
MEDICAL UNIVERSITY OF SOUTH CAROLINA
CHARLESTON, SC 29425

WELLS, BRIAN JAY, MD, MS, PHD, BS
ASSOCIATE PROFESSOR
DEPARTMENT OF BIOSTATISTICAL AND DATA SCIENCE
WAKE FOREST SCHOOL OF MEDICINE
MEDICAL CENTER BOULEVARD
WINSTON-SALEM, NC 27157

XIE, ANPING, PHD
ASSISTANT PROFESSOR
ARMSTRONG INSTITUTE FOR PATIENT SAFETY
DEPT. OF ANESTHESIOLOGY & CRITICAL CARE MEDICINE
JOHNS HOPKINS SCHOOL OF MEDICINE
BALTIMORE, MD 21202

XIE, YANG, PHD, MD, MPH *
PROFESSOR
DEPARTMENT OF POPULATION AND DATA SCIENCES
DEPARTMENT OF BIOINFORMATICS
UNIVERSITY OF TEXAS SOUTHWESTERN MEDICAL CENTER
DALLAS, TX 75390

YEN, PO-YIN, PHD, RN
ASSOCIATE PROFESSOR
SCHOOL OF MEDICINE, INSTITUTE FOR INFORMATICS
GOLDFARB SCHOOL OF NURSING
WASHINGTON UNIVERSITY IN ST. LOUIS
BARNES-JEWISH COLLEGE, BJC HEALTHCARE
BARNES-JEWISH COLLEGE, BJC HEALTHCARE
ST LOUIS, MO 63110

SCIENTIFIC REVIEW OFFICER

APONTE, BORIS, PHD
DIVISION OF SCIENTIFIC REVIEW
OFFICE OF EXTRAMURAL RESEARCH, EDUCATION
AND PRIORITY POPULATIONS
AGENCY FOR HEALTHCARE RESEARCH AND QUALITY
ROCKVILLE, MD 20857

EXTRAMURAL SUPPORT ASSISTANT

LEWIS-MURRAY, PAULA
PROGRAM ANALYST
DIVISION OF SCIENTIFIC REVIEW (DSR)
OFFICE OF EXTRAMURAL RESEARCH, EDUCATION
AND PRIORITY POPULATION
AGENCY FOR HEALTHCARE RESEARCH AND QUALITY
ROCKVILLE, MD 20857

* Temporary Member. For grant applications, temporary members may participate in the entire meeting or may review only selected applications as needed.

Consultants are required to absent themselves from the room during the review of any application if their presence would constitute or appear to constitute a conflict of interest.
