# Supplementary material for: A Digital Communication Intervention to Support Older Adults and Their Care Partners Transitioning Home After Major Surgery: Protocol for a Qualitative Research Study
Source: JMIR Res Protoc. 2024 Aug 28;13:e59067. doi: 10.2196/59067 (PMC11391150; doi:10.2196/59067)
Supplement: Multimedia Appendix 2 [file resprot_v13i1e59067_app2.docx]

**Question Script Guide for the Patient Interview**

**I. Understanding recovery needs (5 min)**

Recovering from surgery can be challenging, especially for patients facing surgery over the age of 65. Often patients will have a care-partner, which is a family member or friend, partner with them during the recovery process to help remember instructions, drive them to appointments or help with daily activities, such as grocery shopping or cleaning.

1. What was your goal for having the surgery?
2. Can you remember how you (and your care-partner) felt preparing for surgery?
3. Tell me how you felt leaving the hospital after surgery?

- Probe: Did you go home? Did you have a visiting nurse? How often? Did you have a care-partner/family to help?

***A. Challenges (15min):***

Tell me about challenges you faced after surgery

- Probe: Did you have any concerns about how your recovery was progressing or concerns for medical or surgical complications?
- Probes:
  1. Did you have challenges related to managing your wounds?
  2. Did you have difficulty managing drains?
  3. Did you have difficulty managing new medications?
- Probe: Did you have any challenges related to getting answers to questions regarding your recovery or communication and follow up with your healthcare team?
- Probe: Did you have challenges that affected your mind (ability to think, memory, or emotions)?
- Probe: Did you have challenges related to your mobility or ability to care for yourself?

Were there any additional challenges **your care partner** faced after surgery?

***B. Solutions (15min):***

What worked in helping you deal with these challenges?

If yes to any probes above…

- Probe: How did you deal with your concerns related to medical or surgical complications?
- Probe: How did you deal with your concerns related to managing your wounds, drains, medications, etc.?
- Probe: How did you deal with the challenges related to healthcare team communication?
- Probe: How did you overcome changes to your mind (ability to think or emotions)?
- Probe: How did you overcome the effect of surgery on your mobility or ability to care for yourself?

Additional probes

- How are you overcoming challenges adapting to your daily life after surgery?
- Probe: Do you think having the surgery will achieve your goal of what you hoped the surgery would do for you (e.g., reduce pain, stop medications, gain more autonomy)?

***C. Resources (15min- if already at 45 minutes, skip this section and go to intervention section):***

What kinds of resources (i.e, tools, educational documents, contacts, monitoring, other supports) were you (and your care partner) provided to assist you in ***preparing for surgery?***

1. Did you use these resources? If not, why not?
2. What kinds of resources or information were most helpful?
3. What resources were unhelpful?
4. What resources do you think would have been helpful to you that you did not receive?
5. **How could we improve and provide important information to you?**

What kinds of resources were you provided to assist you (and your care partner) in ***recovering after surgery?***

1. Did you use these resources? If not, why not?
2. What kinds of resources or information were most helpful?
3. What resources were unhelpful?
4. What resources do you think would have been helpful to you that you did not receive?
5. **How could we improve and provide important information to you?**

Probes for specific resources:

1. Tell me about the information your surgical team shared with you ***after your surgery*** about what surgical recovery would be like?
2. Tell me about information your surgical team shared with you about recognizing and dealing with complications?
3. Tell me about information your surgical team shared with you about your medications or caring for surgical wounds/incisions, drains?
4. What information did they share with you regarding how surgery recovery  might impact  your mind (memory, thinking, emotions)?
5. Tell me about the information your surgical team shared with you about your physical recovery and caring for yourself?
6. Tell me about information your surgical team shared with you about how to achieve your outcomes/goals?
7. Tell me about how you’re most comfortable communicating with clinical staff and being provided information (e.g., Digital recordings, Virtual visits, In person visits, Combination)
8. Who would you like to see provide information prior to surgery? (e.g., surgeon, nurses, anesthesiologists, office staff, past patients)

**II. Communication- what communication needs do participants have (5 min)**

1. When preparing for surgery what individuals, teams or programs do you feel are the most important to communicate with?
2. When recovering from surgery what individuals, teams or programs do you feel are the most important to communicate with?
3. What form of communication do you think works best for connecting with the healthcare team?
4. What makes this communication most effective?
5. What are the challenges in communication with the healthcare team?

**III. Intervention narrative - have participants describe their “ideal” (15 min)**

As part of our research, we hope to develop a technology-based program, such as a web-based or mobile-phone based program that could be used on a desktop computer or smartphone to help older adults undergoing major surgery, and their care-partner,  before, during, and after their procedure. This intervention would likely consist of a program that will ask you a series of questions, provide guidance based on your concerns, and a way to get in touch with clinicians. You could participate alone or with a family member.

1. How might/would an intervention like this impact your recovery?
2. In your ideal world, what would you want this program to include?
3. What type of technology do you feel most comfortable using? (e.g., smartphone based or desktop computers)?
4. What might prevent you from using the program to assist in recovery?
5. Would you like to have a family member or care-partner have access to your app and be able to participate in using it?
6. In what ways could this program benefit a care-partner?
7. What are the things that would be important to include in the program to make it work best for the care-partner?
8. What might prevent a care-partner from using a mobile app like this?

**IV. Wrap up**

1. Is there anything else that we did not ask that you would like to share?

**Thank you for participating.**
