# Supplementary material for: A Digital Communication Intervention to Support Older Adults and Their Care Partners Transitioning Home After Major Surgery: Protocol for a Qualitative Research Study
Source: JMIR Res Protoc. 2024 Aug 28;13:e59067. doi: 10.2196/59067 (PMC11391150; doi:10.2196/59067)
Supplement: Multimedia Appendix 4 [file resprot_v13i1e59067_app4.docx]

**Qualitative Script for Clinicians Taking Care of Older Adults Undergoing Major Surgery**

**I. Challenges/Solutions:**

Recovering from surgery can be challenging, especially for older patients. I want each of you to consider a recent patient you cared for who was over 65 years old, or relatively frail, with a care partner who was involved in their recovery after surgery.

For this discussion when we refer to care-partner we think of a family member or a loved one who partners in care, not a professional person providing care

Let’s get started:

1. What are some major challenges that older adults face when considering surgery or heading into surgery? What about recovering from surgery?
   1. (if silent) Examples of challenges are physical limitations, cognitive limitations, financial issues, communication issues.
   2. How have you seen patients address these challenges successfully on their own?
      1. When they get assistance, what kind of help/services have they engaged to assist them?
      2. What role have you seen care partners play in a patient’s preparation for surgery or their recovery?
2. What types of support (social, nutritional, physical, emotional, among others) do you think might help address some of the challenges encountered by older patients?

Probes:

1. Which supports could help encourage patients to
   1. adhere to instructions?
   2. improve nutrition?
   3. work on their mobility and daily activities
   4. address mental health issues?
2. Once at home, what specific types of support might the dyad of a patient and their care-partner require after surgery during the recovery process?

Probes:

- 1. How might care-partner needs differ from patient needs?
  2. What specific communication or educational support might be of value?

1. Now consider an older patient and care-partner dyad who does not speak English. How are the challenges they face different?
   1. How could digital technology facilitate communication for these patients and care-partners?

**II. Intervention narrative - have participants describe their “ideal”**

We are going to change course now for the last part. As part of our research, we plan to develop a digital intervention to help older adults undergoing major surgery and their care-partners before, during, and after their procedure. This intervention could be accessible through a web-based application, or a smartphone, and would consist of a series of questions/ evaluations aiming to understand the specific needs and challenges that both the patients and the care partners are facing to tailor management recommendations and reminders for the patient/care-partner dyad. The patient could use this alone, or with a care-partner who will access an interconnected account.

1. How could an intervention like this impact **patient preparation** for surgery?
2. How could this impact **patient recovery**?
3. What evaluations or assessments would you find useful to integrate in the intervention?
4. Now let’s take a couple of minutes to look at a simplified version of a prototype assessment tool that we could integrate into the intervention (show on the screen the image below) [Ask the participant to think of the 3 questions below while they review the tool and then those 3 questions will be discussed]
   1. Are the domains the right ones?
   2. Are there evaluations or assessments that are not clear?
   3. Are there evaluations or assessments you would add? Remove?


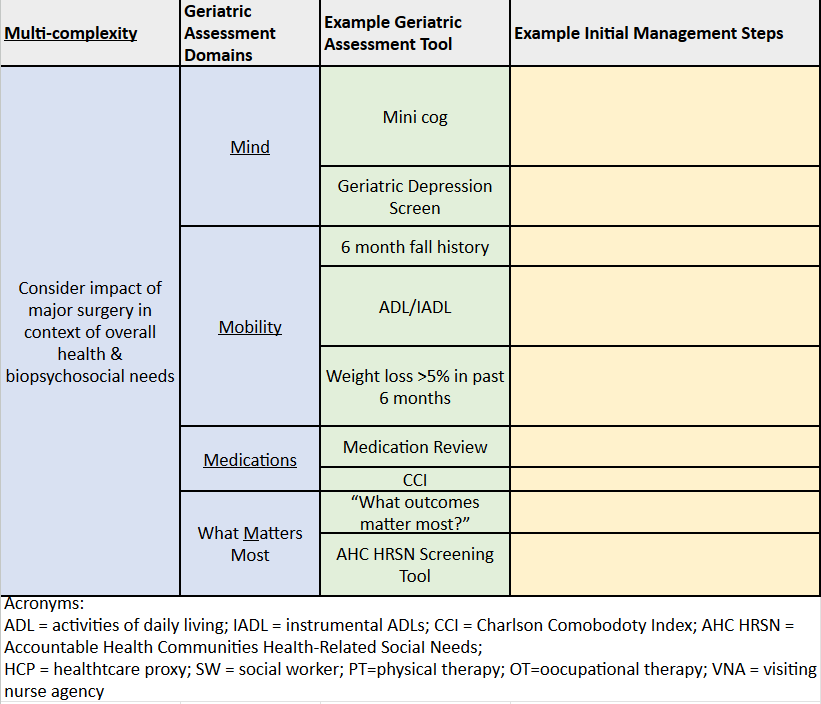


1. Now let’s look at a version of the prototype assessment tool that includes management steps (show on the screen the image below) [Ask the participant to think of the 3 questions below while they review the tool and then those 3 questions will be discussed]
   1. Are the management steps the right ones?
   2. Are there management steps missing?


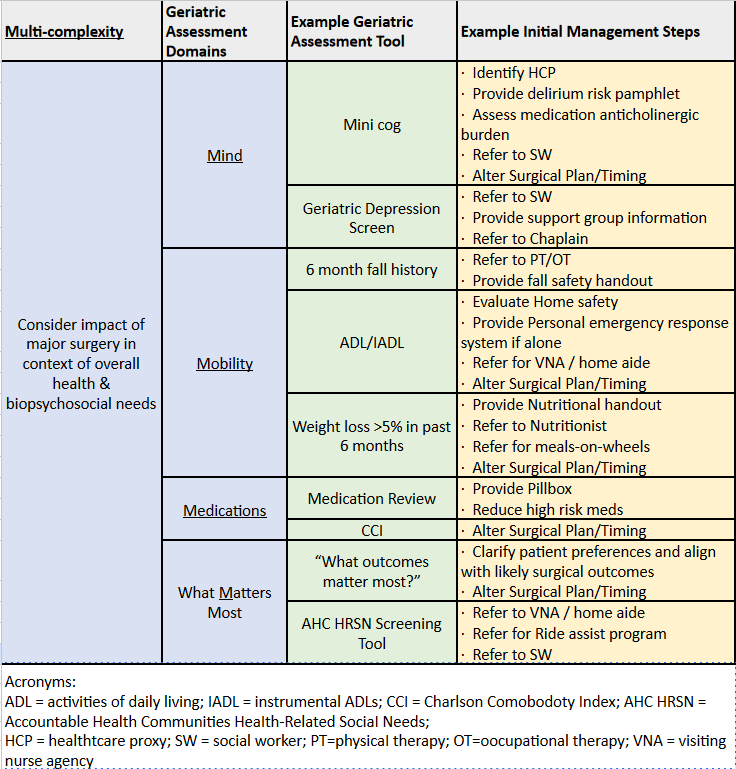


1. What are other essential features, other than the assessment tool, that would have to be integrated into the intervention?
2. Which issues identified in the assessment/evaluation are appropriate to address (in management steps) using a digital/web-based application?
   1. How would this work best?
   2. Which should not be addressed in a digital or web/based application?
   3. Are those considered similar for the care-partner using the intervention?
3. What would be the biggest barriers to implementing this digital intervention (e.g., assessment, management steps, etc.) in your clinic?
   1. In your ideal world, who would review the data received from the patients and caregivers from the digital intervention, who would take the primary role in connecting with patients and care-partners regarding management steps when needed, etc.?
4. What outcomes might be improved with this type of intervention?
5. How can this be designed to ensure the benefit of the information outweighs the burden of review?
6. Knowing time is always a constraint (patient care, charting, notes, etc.), how do you perceive this would overall impact the time spent on doing patient follow-up compared to current standard of care?

**WRAP UP QUESTION**

Anything important you would like to add that was not covered during this focus group?

**Thank you for participating.** [*Instruct to press the red hang-up button.*]
